# Exercise frequency affects morphology of aortic calcium deposits in female hyperlipidemic mice as determined by ^18^F‐NaF PET

**DOI:** 10.14814/phy2.70322

**Published:** 2025-04-19

**Authors:** Nora Safvati, Sophia Kalanski, Andy Hon, Stuti Pradhan, Mimi Lu, Linda L. Demer, Yin Tintut

**Affiliations:** ^1^ Department of Medicine University of California Los Angeles California USA; ^2^ Department of Bioengineering University of California Los Angeles California USA; ^3^ Department of Physiology University of California Los Angeles California USA; ^4^ Department of Orthopaedic Surgery University of California Los Angeles California USA

**Keywords:** calcification, exercise, hyperlipidemia, PET/CT

## Abstract

While exercise is known to benefit cardiovascular health, the optimum regimen, in terms of both speed and frequency, remains unclear, especially for those with existing calcific atherosclerosis. We previously found that, in atherosclerotic female mice, lower speed, but not higher speed, treadmill running had a beneficial effect on the morphology of aortic calcium mineral deposits, as determined by ^18^F‐NaF PET imaging, where ^18^F‐NaF tracer uptake reflects mineral surface area, which, in turn, reflects risk. To determine optimal exercise frequency at the lower speed, ^18^F‐NaF tracer uptake and histochemical analysis of alkaline phosphatase, calcium mineral, and CD68 in the aortas of aged *Apoe* ^*−/−*^ mice exercising 0, 3, or 5 days/week were performed. Images were acquired at baseline and at the end of the study. Although by histochemistry, all 3 groups had similar levels of osteoblastic differentiation and similar numbers of aortic calcium deposits, ^18^F‐NaF tracer uptake increased significantly over the study duration in the 0‐ and 3‐days/week. groups but not in the 5‐days/week. group. Calcification also had a significant negative correlation with macrophage infiltration in the 5‐days/week. group. In summary, the findings suggest that greater frequency running regimens alter aortic calcification in ways that may provide better cardiovascular benefits.

## 1 INTRODUCTION

Physical activity is associated with a lower risk for cardiovascular events and mortality (Kelley & Kelley, 2006). Yet paradoxically, compared with less active controls, elite endurance athletes have a significantly greater prevalence of coronary artery calcification (CAC) (Aengevaeren et al., 2017), which is generally associated with a greater risk (Arnson et al., 2017). The U‐shaped relationship between physical activity level and cardiovascular events in humans (Armstrong et al., 2015) suggests the need to optimize exercise regimens with respect to both intensity and frequency.

Some clinical studies have shown that high‐risk plaques tend to have clusters of multiple, small (1–3 mm) calcium deposits (Ehara et al., 2004), which have more surface area than contiguous calcification. Theoretical analysis suggests that the risk of plaque rupture and intramural hemorrhage, which lead to cardiovascular events, increases with the surface area of calcium deposits due to compliance mismatch and debonding (Barrett et al., 2019). Given the known adsorption and covalent binding of fluoride to hydroxyapatite mineral (White et al., 1988), the surface area of calcium deposits can now be quantified using PET imaging with ^18^F‐NaF radiotracer, as demonstrated by Irkle and colleagues using calcified human carotid atherosclerotic plaques (Irkle et al., 2015). Thus, ^18^F‐NaF tracer uptake may be a more relevant biomarker of plaque rupture risk than total calcium content, such as by Agatston calcium scores.

In our recent study, we tested varying treadmill‐running speeds, 0, 12.5 m/min, and 18.5 m/min in hyperlipidemic mice and found that a relatively low treadmill‐running speed (12.5 m/min) reduced aortic ^18^F‐NaF PET tracer uptake (Hon et al., 2023). Since the frequency of exercise needed to achieve this benefit is not known, in this study, we tested different frequencies of exercise for effects on the surface area of calcific plaque in hyperlipidemic mice.

## 2 MATERIALS AND METHODS

### 2.1 Mice and treadmill exercise regimen

Experimental protocols were reviewed and approved by the Institutional Animal Care and Use Committee of the University of California, Los Angeles. Since there were no effects of exercise on the progression and mineral surface area of aortic calcification in male mice (Hon et al., 2023), only female mice were used in this study. *Apoe* ^−/−^ mice (*n* = 44, ~8‐month‐old, retired breeders on C57BL/6 background; Jackson Laboratory) were placed on a “Western” diet (21% fat and 0.2% cholesterol; Inotiv) to induce baseline aortic calcification for ~2 months. Before the start of the experiment, the mice were acclimated to the treadmill (Columbus Instruments, Exer‐3/6 Animal Treadmill Rodent, 6‐Lane) for 3–4 days followed by a one‐time, 10‐min test session for running capacity at 12.5 m/min. Three mice that did not tolerate the test were excluded. The remaining mice were divided into 3 groups (13 mice/group)–control (no treadmill), 3‐day (3 days/week), or 5‐day (5 days/week)–and subjected to treadmill running for 5 weeks. The speed and duration of treadmill runs (0° slope, no electric shock stimulation) for the 3‐day and 5‐day groups were kept constant at 12.5 m/min and 30 min. To reduce any potential confounding effects of the Western diet, all mice were returned to the standard diet (#7013, Inotiv) for the duration of the exercise regimen. The mice were euthanized by isoflurane anesthesia followed by cardiectomy. Two mice in the 3‐day group were excluded from the analysis due to premature death.

### 2.2 Serial in vivo ^18^F‐NaF microPET/microCT imaging and analysis

**FIGURE 1:**
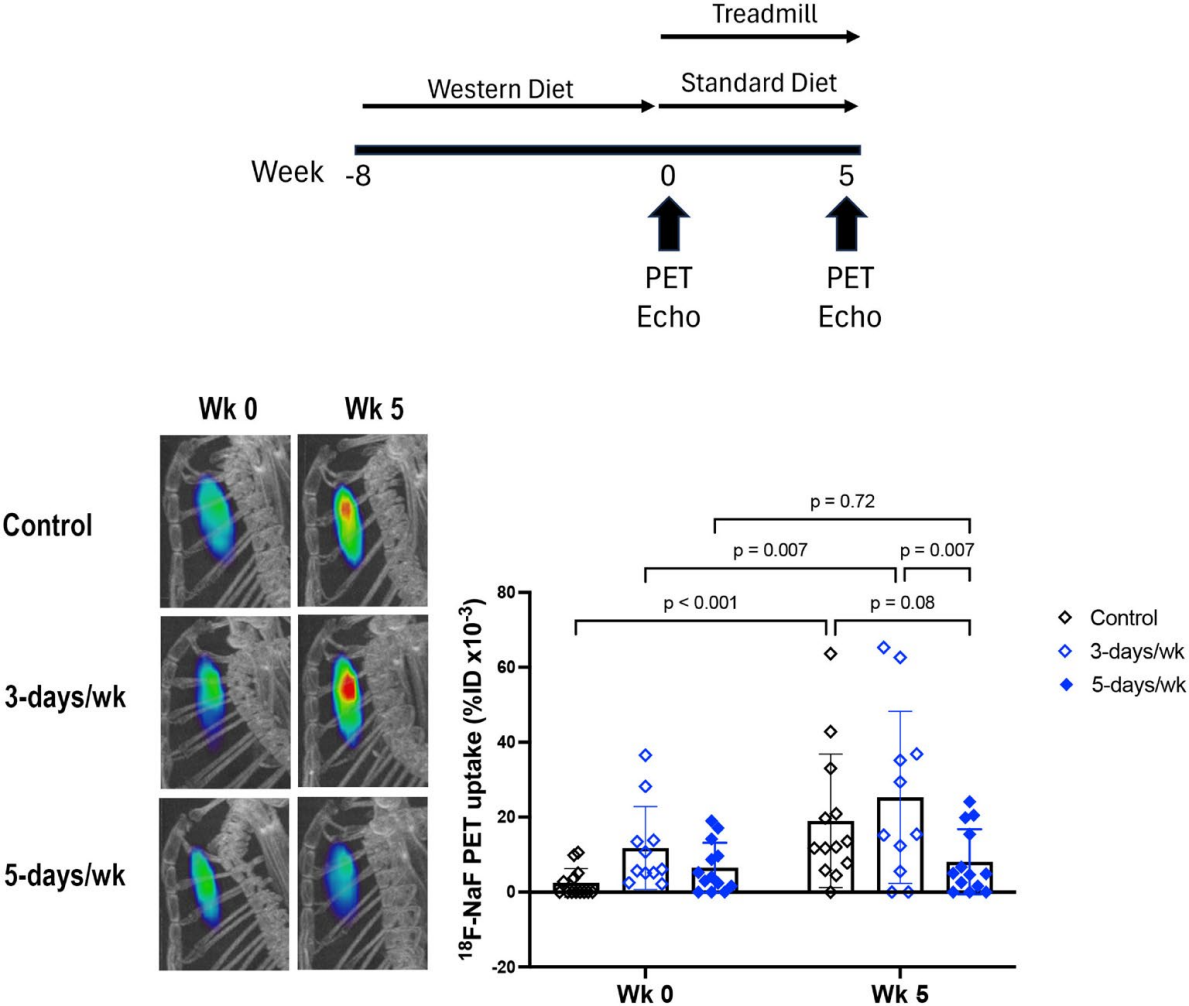
MicroPET imaging of aortic calcification. (Top) Schematic diagram of experimental design. (Bottom) Aortic ^18^F‐NaF tracer uptake, assessed by microPET imaging. *N* = 11–13/group.

Fused ^18^F‐NaF microPET/microCT images were acquired at weeks 0 and 5 at the Preclinical Imaging Facility of the Crump Institute for Molecular Imaging at the California NanoSystems Institute at UCLA (Figure 1). The imaging and analysis protocols were described previously (Hsu et al., 2018). Briefly, mice were injected with ~90 μCi ^18^F‐NaF via the tail vein. One hour post‐injection, the mice were anesthetized and imaged in the microPET/microCT scanner (GNEXT). ^18^F‐NaF tracer was synthesized in an on‐site cyclotron at the UCLA Crump Institute for Molecular Imaging in the California NanoSystems Institute.

Images were analyzed using AMIDE software (Loening & Gambhir, 2003). PET quantification for aortic calcification was performed from an isolated volumetric ROI encompassing parts of cardiac and aortic regions with a minimum ^18^F‐NaF isocontour threshold of 2% injected dose per cubic centimeter (%ID/cc). The mean threshold of background ^18^F‐NaF tracer uptake, measured at the cardiac silhouette of four mice, was 0.8 %ID/cc. The total PET tracer uptake was determined from values of mean density and volume. For lumbar bone density, microCT quantification was performed by geometric box region‐of‐interest (ROI) analysis encompassing the third lumbar vertebra (L3) with a threshold of 1000 Hounsfield Units (HU), and the mean density of HU was used for the comparative analysis.

### 2.3 Histochemical and immunohistochemical analysis

Hearts, including the aortic root, were isolated at euthanasia, embedded in OCT, and sectioned at approximately 5–10 μm thickness. Histochemical staining was used to assess calcification by Alizarin red (#AC400480250; Fisher Scientific, Canoga Park, CA), and osteoblastic differentiation by alkaline phosphatase activity (detection buffer, #1585762, NBT/BCIP stock solution, #1681451; Boehringer Mannheim, Germany), and oil red O (#O‐0625; Millipore Sigma, St. Louis, MO), as previously described. (Li et al., 2015; Xian et al., 2021) Immunohistochemistry was performed using antibody to CD68 (1°Ab, #97778S; Cell Signaling Technology, Danvers, MA; 2°Ab, #BA‐1000; Vector Laboratories, Newark, CA). Cross‐sectional areas of the latter two were quantified using a custom‐designed MatLAB (*ver*. R2015a) code and normalized to total vascular medial layer tissue area determined by NIH ImageJ.

### 2.4 Echocardiography

Mice were anesthetized (3.0% isoflurane for initiation and 1.5%–2.0% isoflurane for maintenance delivered via nose cone), and M‐mode and tissue Doppler echocardiography of the left ventricle were performed using a VisualSonics Vevo 3100 equipped with a 30‐MHz linear transducer. Images were acquired at weeks 0 and 5.

### 2.5 Statistical analysis and blinding

Values are expressed as mean ± SD. Statistical analysis was performed with Prism software (GraphPad, v. 9.4.1). A paired *t*‐test or two‐way ANOVA followed by Holm‐Sidak post‐hoc analysis was used for >3 groups. A *p* value ≤0.05 was considered statistically significant. The investigators were partially blinded. The investigator who analyzed the data knew the groups but did not know the expected outcome.

## 3 RESULTS

### 3.1 Effects of exercise frequency on content and morphology of aortic calcification

Results showed that the surface area of calcium mineral deposits in the aorta, measured by ^18^F‐NaF PET tracer uptake, was differentially affected by exercise frequency. The surface area increased significantly over the study period in control mice (from 2.5 ± 3.8 to 19 ± 18 %ID × 10^−3^/cc, *p* < 0.001 Figure 1). It also increased significantly over the study period in mice that engaged in treadmill running for 3 days/week (from 12 ± 11 to 14 ± 20 %ID × 10^−3^/cc, *p* < 0.007, Figure 1). In contrast, in the mice that engaged in treadmill running 5 days/week, the surface area showed no significant change over the study period (from 6.5 ± 6.6 to 1.6 ± 7.5 %ID × 10^−3^/cc, *p* = 0.72, Figure 1).

**FIGURE 2:**
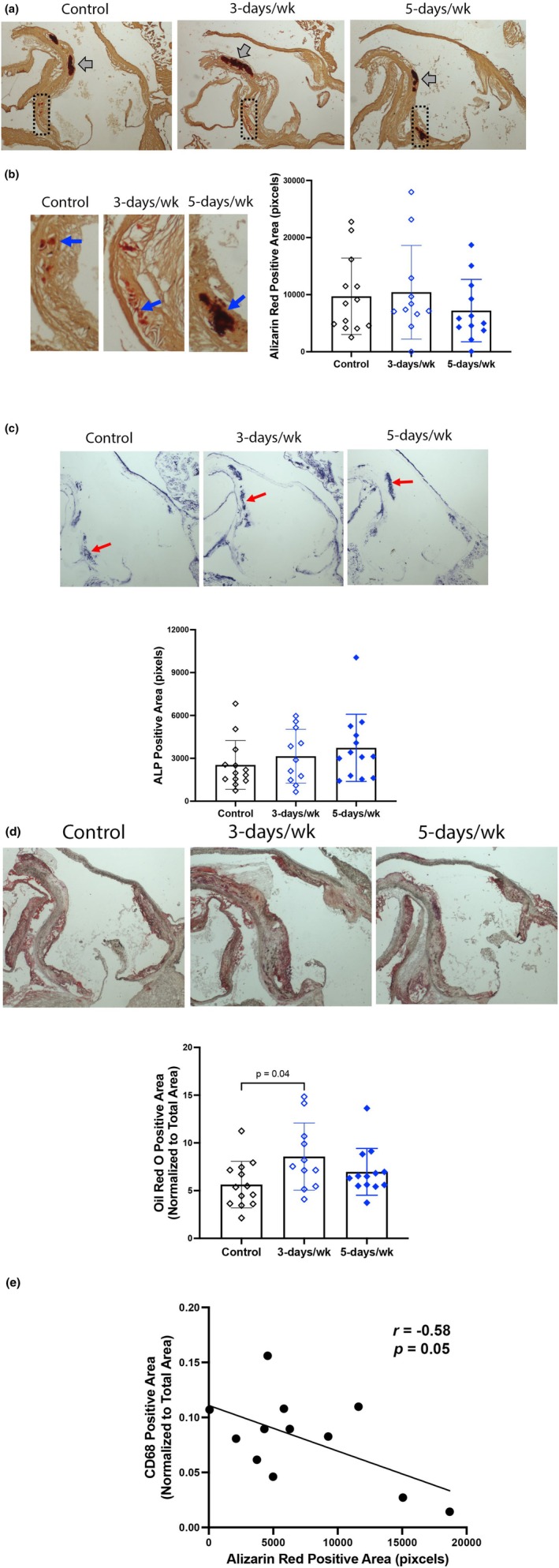
Histological assessment of aortic roots. (a, b) Calcification was assessed by Alizarin red staining *N* = 11–13/group. Gray arrows denote dense, possibly coalesced, calcium deposits. Magnification: 2×. The rectangular dotted outlines indicate the locations for the close‐ups in (b). Control and 3‐days/week groups also have regions of speckled calcium deposits (blue). (c) Osteoblastic differentiation was assessed by alkaline phosphatase histochemical staining (red arrows). *N* = 11–13/group. (d) Atheromatous lesion area was assessed by oil red O histochemical staining (red arrows). Oil red O positivity in the adventitial layer was not included in the calculation. *N* = 11–13/group, magnification: 2×. (e) Correlation of histochemical calcification and infiltration of CD‐68 positive cells, such as macrophages, in mice exercised 5‐days/week. *N* = 12.

To determine whether total calcification was also affected, calcium mineral content was analyzed by histochemical staining on longitudinally sectioned aortic roots. Total calcification by Alizarin red staining was similar among the control, 3‐days/week, and 5‐days/week groups (10 ± 7 vs. 10 ± 8 vs. 7 ± 5 kilopixels, respectively; Figure 2a,b), suggesting that total aortic calcification was not affected. Of note, calcium mineral (gray and blue arrows) occurred as both large contiguous deposits and clusters of small deposits. Similarly, osteoblastic differentiation by alkaline phosphatase activity was similar among the control, 3‐days/week, and 5‐days/week groups (3 ± 2 vs. 3 ± 2 vs. 4 ± 2 kilopixels, respectively; Figure 2c). To test whether lipid‐laden atheromatous lesion area related to the surface area was affected, aortic root sections were stained with oil red O. Interestingly, the 3‐days/week group had significantly greater lipid‐positive lesion area than controls (9% ± 4% vs. 6% ± 2%; Figure 2d), suggesting that exercise frequency has differential effects on lipid versus calcium mineral. Macrophage infiltration in the aortic root, assessed by CD68 immunohistochemistry, showed similar areas of positivity among the three groups (8% ± 3% vs. 11% ± 3% vs. 8% ± 4%, respectively). In our qualitative analysis, we found areas of aortic calcification were surrounded by or close to areas of CD68 immunopositivity, consistent with previous work (Burgmaier et al., 2018). Quantitative analysis showed a significant negative correlation between calcification and macrophage infiltration in the mice exercised 5‐days/week but not in control or 3‐days/week (Figure 2e).

### 3.2 Effects of exercise frequency on cardiac structure and function

Echocardiography was performed at weeks 0 and 5. As shown in Table 1, left ventricular mass and wall thickness were significantly reduced in mice in the 5‐days/week group compared with control and 3‐days/week groups. Cardiac function was not significantly altered by exercise frequency in any of the groups.

**TABLE 1:** Effects of treadmill frequency on echocardiographic parameters.

Parameters	Control (*n* = 12)		3‐day (*n* = 11)		5‐day (*n* = 12)	
	Week 0 mean ± SEM	Week 5 mean ± SEM	Week 0 mean ± SEM	Week 5 mean ± SEM	Week 0 mean ± SEM	Week 5 mean ± SEM
Ejection fraction (%)	57 ± 3	56 ± 2	56 ± 4	59 ± 3	58 ± 3	59 ± 2
Fractional shortening (%)	30 ± 2	29 ± 1	29 ± 3	31 ± 2	30 ± 2	31 ± 2
Diameter, s (mm)	2.5 ± 0.1	2.6 ± 0.1	2.4 ± 0.1	2.4 ± 0.2	2.5 ± 0.1	2.3 ± 0.1
Diameter, d (mm)	3.5 ± 0.1	3.7 ± 0.1	3.4 ± 0.1	3.4 ± 0.2	3.5 ± 0.1	3.3 ± 0.1
LV mass (corrected, mg)	98 ± 4	99 ± 7	96 ± 6	86 ± 5	102 ± 3	81 ± 3^a^, ^b^
LVAW, s (mm)	1.5 ± 0.1	1.5 ± 0.1	1.4 ± 0.1	1.4 ± 0.1	1.6 ± 0.1	1.4 ± 0.1^c^
LVAW, d (mm)	1.07 ± 0.04	1.03 ± 0.06	1.05 ± 0.07	0.99 ± 0.05	1.08 ± 0.05	0.94 ± 0.05^c^
LVPW, s (mm)	1.13 ± 0.04	1.10 ± 0.04	1.22 ± 0.05	1.17 ± 0.05	1.27 ± 0.08	1.11 ± 0.05^d^
LVPW, d (mm)	0.81 ± 0.03	0.77 ± 0.03	0.88 ± 0.03	0.82 ± 0.05	0.97 ± 0.08	0.79 ± 0.04^e^

Abbreviations: Diameter, d, diameter, diastole; Diameter, s, diameter, systole; LV Mass, left ventricular mass; LVAW, d, LV anterior wall, diastole; LVAW, s, LV anterior wall, systole; LVPW, d, LV posterior wall, diastole; LVPW, s, LV posterior wall, systole.

^a^ *p* = 0.01 versus control (Week 5).

^b^ *p* <0.001 versus 5‐day (Week 0).

^c^ *p* = 0.05 versus 5‐day (Week 0).

^d^ *p* = 0.02 versus 5‐day (Week 0).

^e^ *p* = 0.001 versus 5‐day (Week 0).

### 3.3 Effects of exercise frequency on skeletal bone mineral density

**FIGURE 3:**
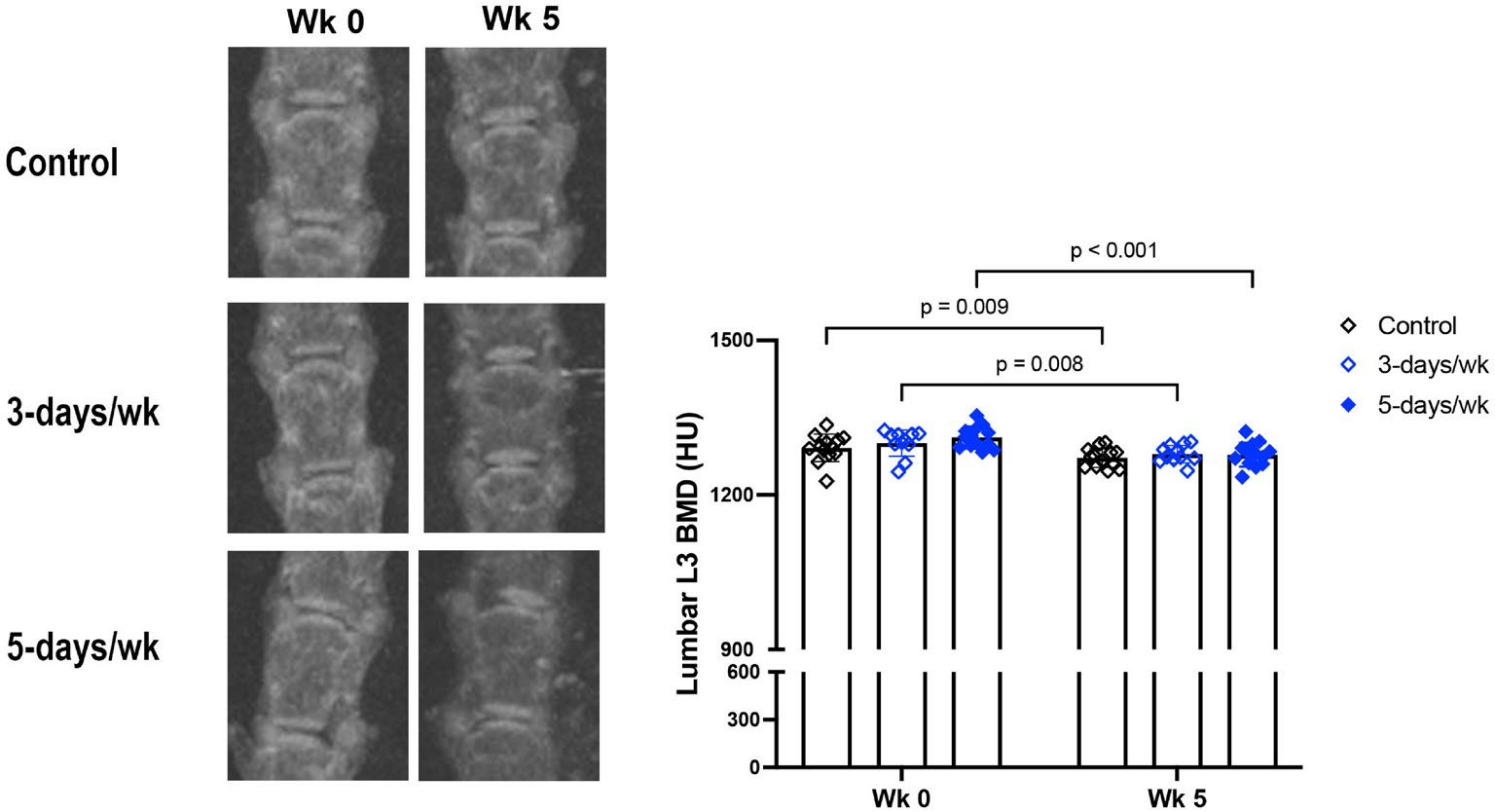
Effects of exercise frequency on skeletal BMD. Skeletal BMD at lumbar vertebrae 3, assessed by microCT imaging. *N* = 11–13/group.

Consistent with our previous findings (Parhami et al., 2001; Pirih et al., 2012; Tintut et al., 2004) of adverse effects of hyperlipidemia on bone density (BMD), results show that lumbar vertebral BMD, assessed by microCT, decreased in all three groups over the 5‐week period (Figure 3). This finding suggests that exercise does not rescue the bone loss associated with hyperlipidemia.

## 4 DISCUSSION

In the present study, we tested whether exercise frequency has an independent effect on aortic calcification and cardiac structure in hyperlipidemic mice with underlying calcific atherosclerosis. Our findings show that aortic calcium mineral surface area, measured by ^18^F‐NaF tracer uptake, was significantly increased in the control and 3‐days/week groups, but not in the 5‐days/week group. In addition, a significant negative correlation was found between calcification and infiltration of CD68‐positive cells, such as macrophages, in the mice exercised 5‐days/week, raising an interesting question about the role of myeloid cells in calcium deposit morphology in the aorta.

Surface area of vascular calcification, which provides valuable information due to its close relation to rupture stress, was assessed by measuring ^18^F‐NaF tracer uptake since the fluoride ions are adsorbed onto the surface of the hydroxyapatite mineral replacing the hydroxyl groups to form fluoroapatite (White et al., 1988). Results of these images differ from those of traditional calcium scans by CT (computed tomography) or microCT because CT provides the total amount (content) of calcium mineral. The findings suggest that the 5‐days/week treadmill‐running regimen induced a change in the morphology of calcium deposits, such as coalescence, that is potentially protective by reducing the surface area subject to compliance mismatch, debonding, and rupture.

By echocardiography, cardiac function (ejection fraction or fractional shortening) was not significantly changed with exercise in any of the three groups. In contrast, cardiac structure was affected: in mice on the 5‐day exercise regimen, left ventricular mass was significantly reduced as was wall thickness. Though unexpected, this cardiac remodeling is consistent with changes seen in human subjects with hypertension following endurance exercise regimens (Baglivo et al., 1990; Turner et al., 2000). Thus, one possible mechanism of this unexpected finding is that the previous dietary hyperlipidemia superimposed on genetic hyperlipidemia and aortic calcification caused hypertension resulting in concentric left ventricular hypertrophy, which would be expected to regress with exercise as observed in human subjects.

With respect to exercise effects on the skeleton, bone density decreased to the same extent in all groups, including mice on the 5‐days/week regimen, consistent with our previous study (Hon et al., 2023). The decrease in bone density is most likely a result of hyperlipidemia, which we found previously to reduce skeletal bone density, apparently overriding the anabolic effects of parathyroid hormone (Huang et al., 2008; Li et al., 2014; Parhami et al., 2001; Pirih et al., 2012), which we previously found to be upregulated with exercise (Hsu et al., 2021). The loss of bone density with hyperlipidemia is supported by human studies showing an association between serum lipid metabolites and osteoporotic fractures (Shao et al., 2024). Although bone loss may also be mediated by hormonal changes with exercise in female athletes (Barrack et al., 2010), the mechanism in the present study is more likely due to hyperlipidemia, since the control mice had a similar degree of bone loss.

Together with our previous study, these findings suggest that, in female mice, an exercise frequency of 5 days/week at a relatively low running speed alters the morphology of calcified lesions in a way that theoretically may confer lower rupture risk in human atherosclerosis. Thus, for women with coronary calcification, this result may provide new insights for designing exercise programs.

## FUNDING INFORMATION

This work was funded by grants from the National Heart, Lung, and Blood Institute (HL137647 and HL151391).

## CONFLICT OF INTEREST STATEMENT

None.

## ETHICS STATEMENT

All animal handling and experimental procedures followed the ethical guidelines and were approved by the Institutional Animal Care and Use Committee of the University of California, Los Angeles.

## Data Availability

The data that support the findings of this study are available from the corresponding author [Y.T.] or LLD, upon a reasonable request.
